# Epidemiologic Features and Risk Factors for Crimean-Congo Hemorrhagic Fever in Dhi Qar Province, Iraq

**DOI:** 10.7759/cureus.61445

**Published:** 2024-05-31

**Authors:** Sarmad H Al-Khafaji, Mohammad H Panahi, Ghazwan Baghdadi, Yadollah Mehrabi, Saeed Hashemi, Ali Delpisheh

**Affiliations:** 1 Department of Epidemiology, School of Public Health & Safety, Shahid Beheshti University of Medical Sciences, Tehran, IRN; 2 Zoonotic Section, Communicable Diseases Control Center, Baghdad, IRQ; 3 Department of Epidemiology, Safety Promotion and Injury Prevention Research Center, School of Public Health & Safety, Shahid Beheshti University of Medical Sciences, Tehran, IRN

**Keywords:** crimean-congo hemorrhagic fever, epidemiology, infectious disease, iraq, dhi-qar, cchf

## Abstract

Background: Crimean-Congo hemorrhagic fever virus (CCHFV) is endemic in Iraq, where recurrent epidemics have been constantly observed during the last five years. The present study aimed to determine the factors associated with Crimean-Congo hemorrhagic fever (CCHF) cases in Dhi Qar province during the year 2022.

Methods: A test-negative case-control design was used to analyze 621 CCHF patients, of which 162 were confirmed and 459 were suspected cases. To identify the confirmed and suspected cases, reverse transcriptase polymerase chain reaction (RT-PCR) was used. Suspected patients whose PCR test results were negative were selected as the control group. Data on potential risk factors for CCHF were collected as existing data for previous years for the same geographical locations in Dhi Qar province. Logistic regression analyses were used to determine the correlation between probable risk factors and confirmed CCHF cases.

Results: The incidence rate of CCHF was 6.8% per 100,000 people. The total number of deaths was 48 for patients with a case fatality rate of 7.7%. The patients' ages ranged from one year to 65 years, with an overall mean ± SD of 36.08 ± 18.29 years. A total of 98.2% of the patients were between 15 and 65 years of age; 58% of the reported patients were male, and the male-to-female ratio was 1.4:1. Additionally, contact with raw meat, animal contact, and tick bite had the highest percentages for CCHF positivity cases.

Conclusions: Male gender, high-risk jobs like housewives, health staff, shepherds, butchers, animal dealers, slaughterhouse workers, veterinary staff, and farmers, tick bites, and contact with raw meat were statistically significant predictors for increasing CCHF incidence in Dhi Qar province during the year 2022.

## Introduction

Crimean-Congo hemorrhagic fever (CCHF) is a contagious illness caused by infection with *Nairovirus*, a member of the *Bunyaviridae* family. It affects both humans and animals [1]. The known distribution of CCHF virus (CCHFV) covers the greatest geographic range of any tick-borne virus and there are reports of viral isolation and/or disease from more than 30 countries across four regions: Africa (Democratic Republic of Congo, Uganda, Mauritania, Nigeria, South Africa, Senegal, and Sudan), Asia (China, Kazakhstan, Tajikistan, Uzbekistan, Afghanistan, Pakistan, and India), Europe (Russia, Bulgaria, Kosovo, Turkey, Greece, and Spain), and the Middle East (Iraq, Iran, Kuwait, Saudi Arabia, Oman, and United Arab Emirates) [1].

The vector for the transmission of CCHF is the *ixodid* tick, especially those belonging to the *Hyalomma* genus. It is also transmitted through contact with infected animals, with most cases occurring among agricultural and slaughterhouse workers. Human-to-human and hospital transmissions are also common due to exposure to infected blood, secretions, unsterilized equipment, etc. The mortality rate for CCHF is nearly 30%, with deaths occurring usually in the second week after infection. The incubation period after initial contact with secretions is usually five to six days, and the maximum can extend to 13 days. However, if the mode of transmission is a tick bite, the incubation period is only one to three days, with a maximum of nine days. The symptoms appear abruptly, presenting with fever, myalgias, dizziness, backache, headache, nausea, vomiting, mood swings, confusion, etc. After that, there follows a period of sleepiness, depression, abdominal pain, hepatomegaly, and several bleeding events. The diagnosis is through enzyme-linked immunosorbent assay (ELISA), antigen detection, virus isolation, and reverse transcriptase polymerase chain reaction (RT-PCR). Management is mainly supportive, with antivirals such as ribavirin proving to be effective [2].

*Hyalomma marginatum* is the main CCHFV vector in Europe, which was detected for the first time in the Netherlands and southern Germany in 2006 [3]. Furthermore, in January 2011, reports of CCHFV associated with *Hyalomma anatolicum* ticks were made for the first time in India [4]. CCHF is endemic across several Middle Eastern countries, including Iraq. In 1979, the disease was first identified in 10 people and has been reported in the nation of Iraq. There were 11 cases in 2010, 10 cases of death in 2018, and 19 confirmed cases with nine deaths (case fatality rate 39%) in 2021.

The Iraqi health authorities reported 212 cases of CCHF to the WHO between 1 January and 22 May 2022, 169 (80%) of which were recorded through April and May alone. Among the 212 patients, 115 were suspected and 97 were confirmed by PCR. Overall, 27 fatal cases occurred (13 of which were confirmed by laboratory tests), and the case fatality rate was 13%. The polymerase chain reaction (PCR) approach was used by the Iraqi Laboratory Center of Public Health to confirm the cases. In confirmed cases, the vast majority of the patients were in direct contact with animals, livestock breeders, or butchers. Just over half of the confirmed patients were 15-44 years old (54%) and male (62%). Approximately 50% of the confirmed cases (48%) were recorded in Dhi Qar, southern Iraq, and the remaining cases were recorded in 12 provinces. The Dhi Qar province consists of rural (42%) and urban (58%) districts, where sheep livestock farming, goats, cattle, and buffaloes are important sources of livelihood, particularly those living in rural areas. Subsistent farming is common in rural areas where animal barns are close to houses and all family members take care of domestic animals. In these regions, CCHF will be transmitted from domestic animals to humans. There is a greater chance that CCHF may expand further, especially in Iraq, on religious holidays such as Eid al-Adha, because many cows and sheep will be slaughtered during these times. Moreover, international cross-border transmission cannot be restricted, leading to increased individual and possible animal passage. During Ramadan, the number of CCHF cases gradually increased, and the geographical spread of the disease expanded to many provinces [5]. The present study aims to determine the factors associated with CCHF cases in Dhi Qar province during the year 2022.

## Materials and methods

Study design

A test-negative case-control design was used to identify confirmed and suspected cases via RT-PCR, and suspected cases whose PCR results were negative composed the control group. This approach has important advantages and efficiently selects cases and controls in the same location using the same case definition, ensuring that they originate from the same source population and reducing potential selection biases. A total of 621 cases, including both confirmed and suspected cases, were recorded to the surveillance system from 1st January to 31st December 2022; these cases included 162 laboratory-confirmed cases and 459 suspected cases. Age, gender, job, laboratory results, clinical characteristics, and outcomes of the suspected and confirmed cases were compared. The data sources were taken from the case investigation forms of all confirmed and suspected cases in the Iraqi CDC of Dhi Qar province. The case investigation form consists of questions divided into four parts. The first part is demographic information and the second part includes questions to assess clinical characteristics and laboratory investigation. The third part is about fate, which includes cured, discharge on their responsibility, death, date of death, and date of discharge from the hospital. The last part is about the epidemiologic investigation at the patient’s home. According to this form, the most important variables needed in the study are mentioned. The jobs of the participants were categorized as low and high-risk groups. In high-risk jobs, individuals exposed to vectors (*Hyalomma* ticks), infected animals, and the blood and tissues of freshly slaughtered livestock, such as housewives, health staff, shepherds, butchers, animal dealers, slaughterhouse workers, veterinary staff, and farmers, are at greatest risk [6]. Low-risk jobs were defined as those in which there is no direct contact with the blood or tissues of freshly slaughtered livestock, such as students, unemployed individuals, and drivers.

Data analysis

Data were analyzed to determine the geographic distribution of CCHF cases and identify any significant risk factors for CCHF transmission. Descriptive statistics were used to summarize and present key characteristics of the study population, CCHF patients, and non-CCHF patients. This includes calculating mean, standard deviation, frequencies, and percentages for relevant variables, such as age, sex, clinical symptoms, and exposure factors. The incidence rates were calculated for the Dhi Qar region in 2022. Univariate analyses were conducted to determine the associations between potential risk factors and the occurrence of confirmed CCHF. Chi-square and T-tests were used to compare the characteristics between confirmed and suspected CCHF patients. Logistic regression analysis was performed to assess the independent effects of multiple risk factors on the odds of developing CCHF. Variables found to be nearly significant (p < 0.2) in the bivariate analyses were included in the model. Adjusted odds ratios and their 95% CIs were calculated to quantify the strength of correlations. P-values less than 0.05 were considered to indicate the statistical significance and SPSS software version 25 (IBM Corp., Armonk, NY) was used for data analysis.

Ethical statement

The protocol of this study was approved by the Ethical Committee of the School of Public Health and Safety (PHS), Shahid Beheshti University of Medical Sciences (SBMU) (Approval ID: IR. SBMU. PHNS.REC.1402.144) and the Iraqi Ministry of Health - Public Health Directorate (Approval ID: 1893).

## Results

The total number of deaths was 48 among all confirmed and suspected cases during the year 2022. Table 1 reveals that there was no statistically significant difference in CCHF positivity between genders (p = 0.724). The highest percentage of CCHF-positive individuals was observed at 15-24 years of age. There was a statistically significant association between age groups and CCHF positivity (p = 0.025). In terms of district, there was no statistically significant difference in CCHF positivity across different districts of Dhi Qar (p = 0.796). There was a statistically significant difference in CCHF positivity among individuals in low-risk and high-risk jobs (p < 0.001), with a higher positivity rate observed for high-risk jobs. Overall, the results revealed that age and job category were associated with CCHF positivity, while gender and district were not significantly associated.

**Table 1:** Distribution of study participants according to sociodemographic and occupational characteristics by laboratory results of CCHF in Dhi Qar, Iraq. * The total of some variables is different because of missing data. ** P-value of Pearson chi-square test for categorical variables or t-test for continuous variables. CCHF: Crimean-Congo hemorrhagic fever.

Parameter		Negative (n = 459) (%)	Positive (n = 162) (%)	Total* (n = 621) (%)	P-value**
Gender	Female	200 (43.6)	68 (42.0)	268 (43.2)	0.724
	Male	259 (56.4)	94 (58.0)	353 (56.8)	
Age (mean ± SD)		35.76 ± 18.92	36.98 ± 16.37	36.08 ± 18.291	0.112
Age group (years)	1-4	6 (1.3)	1 (0.6)	7 (1.1)	0.025
	5-14	29 (6.3)	2 (1.2)	31 (5.0)	
	15-24	112 (24.4)	50 (30.9)	162 (26.1)	
	25-44	179 (39.0)	56 (34.6)	235 (37.8)	
	45-64	83 (18.1)	40 (24.7)	123 (29.8)	
	>=65	50 (10.9)	13 (8.0)	63 (10.1)	
Districts	North	164 (35.7)	58 (35.8)	222 (35.7)	0.796
	Middle	179 (39.0)	67 (41.4)	246 (49.6)	
	South	116 (25.3)	37 (22.8)	153 (24.6)	
Job category	Low-risk jobs	277 (60.3)	70 (43.5)	347 (56.1)	<0.001
	High-risk jobs	180 (39.4)	91 (56.5)	271 (43.9)	

Table 2 provides the results of laboratory testing for CCHF and its association with various clinical symptoms. Fever was common among the individuals who tested positive for CCHF (p = 0.007), while the individuals who experienced bleeding at the injection site were more likely to test positive for CCHF than those who did not (p = 0.015). Bleeding of orifices (revealed bleeding from orifices such as the nose, mouth, and gastrointestinal tract) was not significantly associated with CCHF positivity (p > 0.05 for all).

**Table 2:** Distribution of study participants according to clinical characteristics and laboratory CCHF results in Dhi Qar, Iraq. * The total of some variables is different because of missing data. ** P-value of chi-square test. GIT: gastrointestinal tract; CCHF: Crimean-Congo hemorrhagic fever.

Outcomes	Negative (n = 459) (%)	Positive (n = 162) (%)	*Total (n = 621) (%)	**P-value
Fever	404 (88.4)	155 (95.7)	559 (90.3)	0.007
Bleeding of injection site	29 (6.3)	20 (12.3)	49 (7.9)	0.015
Bleeding of orifices	39 (8.5)	14 (8.6)	53 (8.6)	0.966
Bleeding of nose	36 (7.9)	11 (6.8)	47 (7.6)	0.667
Bleeding of mouth	26 (5.7)	16 (9.9)	42 (6.8)	0.069
Bleeding of GIT	5 (1.1)	1 (0.6)	6 (1.0)	0.599
Ecchymosis	34 (7.4)	19 (11.7)	53 (8.6)	0.094
Total bleeding	97 (21.1)	43 (26.5)	140 (22.5)	-

The odds ratio of the 15-24 years age group was 0.27 (95% CI: 0.06, 1.26) times greater for the risk of CCHF positivity than for the one to four years age group (p = 0.095). The odds ratio for males was 1.07 (95% CI: 0.74, 1.53) times greater than that for females (p = 0.724). The individuals in the central and southern districts had ORs of 1.06 (95% CI: 0.70, 1.59) and 0.90 (95% CI: 0.56, 1.45) for positive CCHF, respectively (p = 0.786, 0.671). Finally, individuals in high-risk jobs had an OR of 2.00 (95% CI: 1.39, 2.88) and a greater risk of positive CCHF than those in low-risk jobs (p < 0.001) (Table 3).

**Table 3:** ORs of demographic characteristics for CCHF patients according to univariate logistic regression analysis. * Odds ratios computed by univariate logistic regression. ** A total of 1.9% of low-risk workers were retired, followed by farmers and soldiers. *** High-risk jobs were housewives, accounting for 38.3% of the positive jobs. Overall, 9.9% of butchers were in the most common job category of contact with raw meat. CCHF: Crimean-Congo hemorrhagic fever.

Demographic	Crude OR (95% CI)*	P-value
Age group (years)		
1-4	(Ref.)	
5-14	0.64 (0.71, 5.80)	0.692
15-24	0.27 (0.06, 1.26)	0.095
25-44	1.72 (0.86, 3.44)	0.128
45-64	1.20 (0.61, 2.37)	0.594
>=65	1.85 (0.90, 3.80)	0.092
Sex		
Female	(Ref.)	
Male	1.07 (0.74, 1.53)	0.724
Districts		
North	(Ref.)	
Middle	1.06 (0.70, 1.59)	0.786
South	0.90 (0.56, 1.45)	0.671
Job category		
Low-risk jobs**	(Ref.)	
High-risk jobs***	2.00 (1.39, 2.88)	<0.001

Patients with CCHF positivity had 2.91 (95% CI: 1.29, 6.53) times more fever than those with CCHF negativity (p = 0.010). In addition, patients with positive CCHF results experienced 2.08 (95% CI: 1.14, 3.79) times more bleeding at the injection site than those with suspected but negative CCHF results (p = 0.017). With respect to bleeding of the orifices, nose, mouth, and gastrointestinal tract (GIT), these outcomes did not show statistically significant associations with CCHF. Finally, the crude OR of ecchymosis was 1.65 (95% CI: 0.91, 2.99) in CCHF-positive patients compared to CCHF-negative patients (Table 4).

**Table 4:** Odds ratio of confirmed CCHF for clinical outcomes according to univariate logistic regression analysis. * Odds ratios of confirmed CCHF patients compared to suspected CCHF patients computed by univariate binary logistic regression. GIT: gastrointestinal tract; CCHF: Crimean-Congo hemorrhagic fever.

Outcome	Crude OR (95%CI)*	P-value
Fever	2.91 (1.29, 6.53)	0.010
Bleeding of injection site	2.08 (1.14, 3.79)	0.017
Bleeding of orifices	1.01 (0.54, 1.92)	0.966
Bleeding of nose	0.86 (0.43, 1.73)	0.667
Bleeding of mouth	1.82 (0.95, 3.48)	0.072
Bleeding of GIT	0.57 (0.07, 4.87)	0.604
Ecchymosis	1.65 (0.91, 2.99)	0.096

Table 5 presents the adjusted odds ratio (AOR), accompanying p-value, and 95% CI derived from the backward likelihood ratio (LR) method. The odds of CCHF among males were approximately 2.39 times greater than those among females (p = 0.007). Individuals in high-risk jobs had approximately 2.11 times greater odds of experiencing CCHF than those in low-risk jobs (p = 0.022). Participants who experienced a tick bite had approximately 2.51 times greater risk of CCHF than those who did not report a tick bite (p < 0.001). Individuals reporting contact with raw meat had approximately 4.25 times greater odds of having CCHF positivity than those without such exposure (p < 0.001). Participants exposed to the presence of rodents had approximately 1.85 times greater odds of CCHF than those without such exposure (p = 0.006). These associations held significant even after adjusting for other variables included in the analysis.

**Table 5:** Selected models of variables by stepwise backward multivariable logistic methods. * Adjusted odds ratio with multivariate logistic regression.

Selected variables		*AOR (95% CI)	P-value
Gender	Female	1	
	Male	2.39 (1.26, 4.52)	0.007
Job category	Low-risk group (Ref.)	1	
	High-risk group	2.11 (1.12, 4.00)	0.022
Tick bite	No (Ref.)	1	
	Yes	2.51 (1.56, 3.05)	<0.001
Contact with raw meat	No (Ref.)	1	
	Yes	4.25 (2.64, 6.86)	<0.001
Presence of rodents	No (Ref.)	1	
	Yes	1.85 (1.19, 2.88)	0.006

## Discussion

The findings of this research are based on a negative case-control analysis of the epidemiology, clinical, and laboratory features of CCHF patients registered in Dhi Qar province. It is hoped that the findings of this study will aid clinicians and healthcare workers (HCWs) in the early detection of fatal cases in endemic regions because they are aware of some of the striking clinical and laboratory characteristics presented by patients and help guide important and efficient management for future outbreaks.

Since the first incidence of CCHF was documented in 1979, Iraq has developed into an endemic region for CCHF. Every year, multiple cases of CCHF are recorded by Iraqi health authorities, but the disease burden increased in the first half of 2022. From 1st January 2022 to 29th May 2022, Iraq registered 212 cases of CCHF. This number is already six-fold greater than that in 2021 when only 19 cases were recorded throughout the year. A total of 54% of the patients were suspected, and 46% were confirmed [7].

In our study, the major cause of CCHF was the high-risk jobs, which were responsible for more than half of CCHF cases (56.5%), while the low-risk jobs were responsible for 43.5%, followed by the 25-44 years old age group (34.6%) and the 15-24 years old age group (30.9%). These findings are consistent with the findings of a previous study in Afghanistan in 2019, which showed that 16-30-year-old individuals were at risk for CCHF (45%), while the high-risk jobs were approximately 50% [8]. Similarly, a study conducted in Kabul, Afghanistan, in 2023 showed that the percentage of people aged 20-30 years was 30.0%, and another study conducted in Turkey in 2021 reported significant results for age groups, which is consistent with our results [9]. Moreover, the percentages of high-risk jobs (such as butchers (23.3), animal dealers (20.0), shepherds (16.6), housewives (16.6), and farmers (10.0)) were inconsistent with our results [10]. Hence, patients in high-risk jobs (housewives) in Iraqi society are highly exposed to raw meat during their daily cooking, and they are in direct contact with other categories that may be exposed to other risk groups according to the Iraqi lifestyle, especially in rural areas where families are crowded.

Our results disagree with the findings of a study conducted about a large outbreak of CCHF in Iraq in 2022 [11]. The incidence rate of CCHF was greater in this study, which included 108 laboratory-confirmed cases of CCHF between 1st January 2022 and 26th June 2022, which differs from our study that included cases for a longer period from 1st January to 31st December 2022, during which 54 confirmed cases increased. This increase in incidence rate may be explained by the increase in hard tick infestations of animals and farms or the increase may have occurred due to the absence of insect control activities in 2020 and 2021 during the coronavirus disease 2019 (COVID-19) pandemic. In addition, there is a lack of awareness about CCHF and its mode of transmission among butchers, farmers, and the community.

The majority of patients had a history of fever as a consequence of CCHF (90.3%), which is comparable with the findings of earlier research in Turkey, which revealed that the prevalence of fever was 90.1% [12]. Their findings confirmed that the prevalence of fever was 98.3% in another study conducted in Kabul, Afghanistan [13]. Moreover, these findings are in line with those of a study conducted in Turkey [14].

Another important predictor was bleeding from the site of injection, which is a clinical feature of CCHF infection, and the proportion of individuals who had this sign (12.3%). These findings agree with those of other studies conducted in 2023 in Iraq (10%) [15].

The odds ratio of our results according to male sex was 1.07 (0.74, 1.53), which is in line with the findings of a study conducted in Bulgaria, which revealed a value of 1.10 (0.64-1.88), both of which were statistically non-significant [16].

To conclude from the findings of our study, the most important epidemiologic risk factors for CCHF were contact with raw meat (AOR: 4.25) and male sex (AOR: 2.39), and the disease is a seasonal problem in this region, similar to other regions of Iraq.

Moreover, the results of the present study revealed that gender and history of tick contact were consistent with those of other studies conducted in Turkey, and the results of this study were statistically significant [9].

The AOR of patients who had a history of tick bites compared to those who did not have such a history of tick bites was 2.51. Farming and being bitten by ticks were determined to be risk factors for CCHF in the multivariate analysis in this study [17].

Strengths & limitations

The strength of this study is that it is the first of its kind in the Dhi Qar governorate to document the determinants of factors that increase the risk of CCHF. Hence, these results should draw policymakers’ attention to the consequences and burden of CCHF on the community in Dhi Qar. A negative case-control design was used to detect CCHF in the patient and control groups. This study describes the largest CCHF outbreak in Iraq since 1979.

One of the study challenges is the absence of complete medical histories and laboratory findings for each patient, which limits comparisons between patients who recovered and patients who died. This comparison highlights the important prognostic factors that increase the chance of recovery.

Furthermore, no information is available regarding the CCHF strains of the confirmed cases, which might have provided greater clarity on the individual’s prognostic factors and the genetic diversity of CCHF in Iraq.

## Conclusions

In this study, a number of factors were found to be statistically significant predictors of CCHF, and the increased incidence of this contagious illness requires effective control strategies. The suitable steps for CCHF prevention and control carried out by the community and the government should be considered. The WHO has a beneficial role in public awareness and disease surveillance for the purpose of disease elimination. The use of insect repellants containing DEET (N, N-diethyl-m-toluamide) is recommended by people in high-risk regions, such as livestock and agricultural workers. It should be encouraged to handle the animals with appropriate gloves. It is important to establish an international collaboration for the monitoring and control of CCHF, particularly in endemic regions. Any animal showing symptoms of infection should be restricted separately, and their contact with people should be restricted. Healthcare workers must be educated about this occupational hazard and how to use proper precautions whenever dealing with patients. Additional studies should prioritize integrative strategies for humans and veterinarians to understand CCHF. Databases created via our study can be used to evaluate the potential risk factors that are correlated with the geographic distribution of CCHF in Dhi Qar province.
